# 

**Published:** 1899-11

**Authors:** 


					﻿HOTICE.— This JOURNAL and THE INTERNATIONAL
JOURNAL OFSURGERI one year for (Rl.OO cash, to new
subscribers, Ho out-of-town checks accepted unless 10c. is
added for cost of cashing.
NOTICE.-This JOURNAL and THE INTERNATION-
AL JOURNAL OF SURGERY one year for $1.00 cash,
to new subscribers. No out-of-town checks ac-
cepted unless lOc. is added for cost of cashing.
NO TICE.— This J O LliNAL and THE IN TEEN A TIONAI
JOURNAL OF SURGERY one year for $1.00 cash, to new
subscribers. No out-of-town checks accepted unless 10c. is
added for cost of cashing.
NOTICE.—This JOURNAL and THE INTERNATIONAL JOUR-
NAL OF SURGERY one year for $1.00 cash, to new subscribers. No
out-of-town checks accepted unless lOc. is added for cost of cashing.
NOTICE,— This JOURNAL and THE INTERNATIONAL
JOURNAL OF SURGERY one year for $1.00 cash, to new
subscribers. No out-of-town checks accepted unless 10c. is
added for cost of cashing.
				

